# Diabetes and Obesity as Independent Risk Factors for Osteoporosis: Updated Results from the ROIS/EMEROS Registry in a Population of Five Thousand Post-Menopausal Women Living in a Region Characterized by Heavy Environmental Pressure

**DOI:** 10.3390/ijerph13111067

**Published:** 2016-11-01

**Authors:** Cosimo Neglia, Alberto Argentiero, Giovanna Chitano, Nadia Agnello, Roberta Ciccarese, Antonella Vigilanza, Valerio Pantile, Domenico Argentiero, Raffaele Quarta, Matteo Rivezzi, Gian Luca Di Tanna, Carolina Di Somma, Alberto Migliore, Giovanni Iolascon, Francesca Gimigliano, Alessandro Distante, Prisco Piscitelli

**Affiliations:** 1 ISBEM (Istituto Scientifico Biomedico Euro Mediterraneo), Brindisi 72100, Italy; argentiero85@gmail.com (A.A.); chitano@isbem.it (G.C.); agnello@isbem.it (N.A.); ciccarese@isbem.it (R.C.); vigilanza@isbem.it (A.V.); pantile@isbem.it (V.P.); argentiero@isbem.it (D.A.); raf.quarta@libero.it (R.Q.); distante@isbem.it (A.D.); 2 IOS, Southern Italy Hospital Institute, Medicina Futura Research, Naples 80100, Italy; matteo.rivezzi@gmail.com (M.R.); priscofreedom@hotmail.com (P.P.); 3 Centre for Primary Care and Public Health, Queen Mary University of London, London E1 4NS, UK; g.ditanna@qmul.ac.uk; 4 IRCCS SDN Istituto di Ricerca Diagnostica e Nucleare, Naples 80133, Italy; cdisomma@unina.it; 5 St. Peter’s Fatebenefratelli Hospital, Rome 00186, Italy; migliore.alberto60@gmail.com; 6 Department of Orthopaedic Surgery and Rehabilitation, Second University of Naples, Naples 81100, Italy; giovanni.iolascon@unina2.it (G.I.); francesca.gimigliano@unina2.it (F.G.)

**Keywords:** diabetes, obesity, environmental factors, osteoporosis, fractures, quantitative ultrasounds

## Abstract

***Objectives*:** We aimed to analyze bone mineralization and the effect of different risk factors for osteoporosis in postmenopausal women. ***Methods*:** We found 4909 postmenopausal subjects within ≥10,000 records from the ROIS/EMEROS (Ionian and Salento Osteoporosis Registry/Euro Mediterranean Registry of Osteoporosis) registry, a population study carried out in an area characterized by heavy environmental pressure between Brindisi and Taranto from 2009 to 2016. All subjects were assessed via phalangeal quantitative ultrasound (QUS) to evaluate their bone mineralization (assessed via amplitude dependent speed of sound (AD-SoS)) and the association between demineralization and the presence of other conditions or risk factors. ***Results*:** Mean age was 64 ± 9.5 years and mean body mass index (BMI) was 28.7 ± 3.5 kg/m^2^. Pearson correlation analyses revealed a negative association between bone mineralization (AD-SoS) and BMI (*p* < 0.001). By using multivariate logistic regression analysis, we observed significant values of odds ratios (ORs) of osteoporosis (adjusted for age, physical activity, and the use of drugs known to increase the risk of fractures) in subjects with diabetes and obesity: 1.39 (confidence interval (CI): 1.05–1.83) and 1.46 (CI: 1.20–1.78), respectively. A statistically significant linear trend of higher ORs of osteoporosis was found for increasing values of BMI. ***Conclusions*:** Our study confirmed the high impact of obesity and type 1 and type 2 diabetes on osteoporosis.

## 1. Introduction

Osteoporosis and fragility fractures represent a growing health problem in developed countries in terms of social costs and increased risk of death, especially in the elderly [1]. Fracture incidence rates are closely related to the ageing of a population, as older people present higher fracture rates than younger subjects with the same bone mineral density [2]. This is due to a lower bone quality and higher tendency to fall. It is estimated that 25% of women aged 80 years and over have already undergone at least one vertebral fracture [3]. 

According to the ESOPO (Epidemiological Study on the Prevalence Osteoporosis in Italy) study, about 5 million Italians suffer from osteoporosis with almost 1.5 million of these subjects being “at high risk” of femoral fracture because they have already experienced one or more vertebral fractures [4]. Hospital costs of femoral fractures in the elderly Italian population account for 1 billion Euros per year [5]. Prevention of osteoporosis is traditionally based on bone measurement tests, aimed to predict the risk of future fragility fractures. Diagnostic criteria for osteoporosis are based on dual energy X-ray absorptiometry (DXA) measures, as performed at the hip or lumbar spine. DXA is considered the gold standard methodology for the diagnosis of bone demineralization. However, other kinds of diagnostic tests are available. Predictive parameter BMD (bone mineral density), which derives from DXA, is used in clinical practice to assess the risk of fracture, even though it is well recognized that other risk factors, such as age and drugs inducing osteoporosis (steroids and others), are independent contributors to the risk of fracture and improve the sensitivity of BMD measurement in identifying patients at risk [6,7].

Quantitative ultrasounds (QUS) at calcaneal (heel) or phalangeal sites represent the most commonly used non-DXA methodologies. This kind of radiation-free test was shown to be user-friendly, cheap, non-invasive, and—most importantly—it is able to discriminate subjects at increased risk of fragility fracture [8]. QUS, developed by Langton et al. in 1984, have been settled for the evaluation of bone quality and skeletal disorders on the basis of various experiments that suggest that the ultrasonic parameters could provide information not only on bone density but also on micro-architecture and elastic properties of bone tissue [9]. The phalangeal QUS methodology was introduced in Europe in 1992–1993, and a series of studies have been performed to validate the method in clinical settings [10,11]. Bone resorption at the proximal phalanx is associated with significant changes in the speed of the ultrasonic signal that passes through the phalanx [12]. Benitez et al. performed a comparison between phalangeal QUS and DXA (measured at total hip and lumbar spine), concluding that phalangeal QUS can be effectively used for the screening of osteoporosis in postmenopausal women [13]. More recently, the assessment of clinical risk factors has become more and more important in the diagnosis of osteoporosis and to select patients for appropriate treatment. 

In this frame, the Euro Mediterranean Bio-Medical Scientific Institute (ISBEM, Brindisi, Italy) has launched a pilot project for the disease management of osteoporosis, which is aimed at the prevention of fragility fractures through the early identification of people at higher risk through the use of phalangeal QUS and the collection of clinical risk factors in the ROIS (Ionian and Salento Osteoporosis Registry), which represents the first section of the EMEROS (Euro Mediterranean Registry of Osteoporosis), acknowledged as an official commitment by the European Commission Action Group on Active and Health Ageing. The registry started in 2009 thanks to a strong cooperation between ISBEM researchers and physicians from the Local Health Authorities (ASL) Brindisi, ASL Taranto, and ASL Lecce (limited to the hospital of Gallipoli, Division of Orthopedics and Traumatology). This first section of the registry is of particular interest because Salento sub-region represents the “oldest” area of Apulia and of entire Southern Italy, as it is characterized by an “ageing index”, very closely reflecting the national average value (which has risen up to 143) [5]. The “ageing index” is an epidemiological indicator that corresponds to the ratio between the number of people aged >65 and the number of children aged 0–14 years old. Therefore, this population could be considered representative of the entire Italian population and could be particularly useful both for case-control analyses and prospective cohort studies, thanks to the monitoring activities carried out within the ROIS registry integrated in the EMEROS. 

In addition, in the study area, there is a strong industrial activity (Brindisi and Taranto) with a high impact on pollution of air, soil, and water and on public health [14]. A study conducted on women living in Taranto showed that the content of polychlorinated dibenzo-*p*-dioxins in human breast milk was 10–40 times higher than legal limit [15]. Furthermore, several epidemiological studies showed an increased mortality/morbidity from respiratory, cardiovascular diseases, and several cancer sites [16,17]. For the years 2005–2009, a higher chronic obstructive pulmonary disease prevalence was found in Brindisi than in other Italian cities [18]. The impact of concentrations of air pollutants in both men and women has been shown in a recent study. The author, in fact, found a positive associations between the presence of pollutants and a risk of osteoporosis; they concluded that high level of air pollutants might increase the risk of osteoporosis by 39% to 89% [19].

In this paper, we present the updated analyses of the ROIS/EMEROS registry focusing on the association between some major clinical conditions (i.e., obesity, dyslipidemia, diabetes, hypertension, cardiovascular diseases, rheumatic diseases, and previous fractures) and bone demineralization in a population living in a specific area characterized by heavy environmental pressure between Brindisi and Taranto.

## 2. Materials and Methods

### 2.1. Subjects

The PR.O.F. (PRrevention of Osteoporosis and Fractures) study was a disease management project for the prevention of fragility fractures carried out at the ambulatorial and hospital centers for the diagnosis and treatment of osteoporosis of Local Health Authority of Brindisi, Taranto and Lecce (limited to the Hospital of Gallipoli, division of Orthopedics and Traumatology). Each subject was provided with adequate information about the privacy policy, and everybody was asked to sign for approval to data processing for the purposes of the study. All personal data was collected in compliance with the Declaration of Helsinki. The study was conducted in accordance with the Local Health Authority (ASL) of Brindisi and Taranto, and the Declarations were approved by

- the General Director of the Local Health Authority (ASL) of Brindisi (Number 3393 of 25 November 2008);
- the General Director of the Local Health Authority (ASL) of Taranto (Number 3240 of 14 September 2009).

In 2016, the Ethics Committee of Lecce approved the study within the European version of the project called EMEROS.

Before the kick-off of the project, ISBEM researchers developed a questionnaire in order to record main clinical information of each patient; the questionnaire includes all the mandatory fields and items of the electronic form developed by the Italian Society of Osteoporosis of Bone Mineral and of Skeletal Disease (SIOMMMS). During the enrollment visit, the weight of all patients was recorded, with a tolerance of 0.5 kg; standing height was measured on the balance stadiometer to the nearest centimeter. Body mass index (BMI) was calculated as weight/height^2^ (kg/m^2^); obesity was defined in the presence of BMI ≥ 30. Each subject was interviewed about frequency of alcohol consumption, cigarette smoking, calcium intake (by milk and dairy products), physical activity, previous fractures, parental history of osteoporotic fractures, age, and cause of menopause. In the interview, the presence of diseases known to be associated with osteoporosis and any other diseases was specifically recorded, as well as any previous or current use of anti-fracture drugs (including vitamin D and calcium supplementations), therapies influencing bone mineralization (i.e., corticosteroid, immunosuppressive, heparin, antiepileptic, antiestrogens, and chemotherapies), and any other current treatment for other diseases. The use of drugs known to increase the risk of falls (i.e., benzodiazepines or hypnotics) was also recorded. Physical activity was assessed with a specific score (0 = no physical activity; 1 = moderate physical activity; 3 = heavy physical activity) developed for clinical records by the Italian National Research Council throughout a systematic review of the available medical literature.

All data were entered into the ROIS registry (Ionian and Salento Osteoporosis Registry) which represents the first section of the EMEROS (Euro Mediterranean Registry of Osteoporosis), acknowledged as official commitment by the European Commission Action Group on Active and Health Ageing. Between February 2009 and June 2016, about 12,000 consecutive patients undergoing bone densitometric examination by QUS were included in the ROIS/EMEROS registry. However, the number of enrolled subjects presenting a complete dataset of clinical variables and QUS measurements needed for the purpose of this study was 6471 (727 men, 4909 postmenopausal women, and 835 pre-menopausal patients). Premenopausal women, men, subjects with extreme BMI values (<18.5 and >50), and thalassemic patients examined at Taranto and Brindisi thalassemia centers were excluded from this study, so the final sample analyzed consisted in 4909 postmenopausal women. 

### 2.2. Ultrasound Measurements

Ultrasound measurements were performed for all subjects using DBM Sonic Bone Profiler 1200 (Igea^®^, Carpi, Italy). This device is based on the transmission of ultrasounds through proximal phalanges (II–V) of the fingers at dominant or non-dominant hand; transmitting and receiving probes are applied to the lateral surface of each finger; the coupling of the probes with the skin is accomplished using simple gel for ultrasound transmission. At each measurement session, the reference values of the patient’s soft tissue were measured by applying the probes to the soft tissue of the hand (“anatomical snuff box”). The measurements were performed by placing probes at the distal metaphysis of the first phalanges, in the proximity of the condyles. The measurement outcomes provided by the device were amplitude-dependent speed of sound (AD-SoS, m/s) and AD-SoS *T*-score, where this latter parameter compares the measured AD-SoS value with the average value of young adults and is expressed as standard deviation (SD). For this device, the specific *T*-score diagnostic threshold discriminating between healthy and osteoporotic people at increased risk of fractures has been set up in a large study carried out on a population of 10,000 subjects and corresponds to −3.2 SD. *T*-score between −3.2 and −1 was defined as osteopenia status [20].

### 2.3. Statistical Analysis

All statistical analyses were performed by using STATA/SE 12 software for Windows (Stata Statistical Software: Release 12. StataCorp LP. College Station, TX, USA). Obesity, dyslipidemia, type 1 and type 2 diabetes, hypertension, cardiovascular diseases, rheumatic diseases, and previous fractures were used as dichotomous variables. 

Continuous data were presented as mean value ± SD; frequency and percentage of dichotomous variables were computed. Baseline characteristics of osteoporotic, osteopenic, and normal postmenopausal subjects were compared via Student’s *t*-test for continuous variables and via linear regression analysis for categorical variables.

Pearson correlation coefficients were calculated to assess the association between AD-SoS and BMI. Linear regression analysis was performed to test the presence of linear trends among different classes of BMI for all the clinical variables.

Multivariate analysis was performed using a logistic regression model to assess the association between bone demineralization status and diabetes, hypertension, cardiovascular diseases, and obesity. Models were adjusted to eliminate the effects of age, physical activity, and the previous or current use of any drug known to be associated with bone demineralization.

Mantel–Haenszel odds ratios of osteoporosis, crude and adjusted for age, physical activity, and the previous or current use of osteoporosis-inducing drugs were calculated to assess the effect of the classes of BMI on the odds of being osteoporotic.

## 3. Results

Table 1 summarizes baseline characteristics of postmenopausal women stratified by their bone mineralization status (osteoporosis, osteopenia, and normal). Mean age was higher in osteoporotic subjects (70.3 ± 8.6) than in osteopenic (62.8 ± 8.3) and normal subjects (54.9 ± 7.1).

Osteoporotic subjects presented a higher mean BMI (29.84; SD 5.56) than osteopenic (28.64; SD 5.19) and normal people (26.71; SD 4.87). AD-SoS mean values were significantly higher (*p* < 0.001) in normal subjects (2087.3 m/s; SD 44.1) than in osteopenic (1967.5 m/s; SD 46.5) and osteoporotic patients (1842.2 m/s; SD 60.7; *p* < 0.0001).

The frequency of obesity, hypertension, type 1 and type 2 diabetes, dyslipidemia, and cardiovascular diseases was significantly higher in osteoporotic subjects (*p* < 0.001). No statistically significant differences were found among the three groups for the presence of rheumatic diseases or in the case of a previous or current use of drugs known to be associated with bone demineralization.

Pearson correlation analysis between AD-SoS and BMI revealed a negative correlation: *r* = −0.2 (*p* < 0.0001).

Table 2 presents the results of multivariate logistic regression analysis between osteoporotic status (AD-SoS *T*-score lower than −3.2 SD) and other conditions such as cardiovascular diseases, type 1 and type 2 diabetes, hypertension, and obesity (BMI ≥ 30). All of these conditions presented odds ratios (ORs) > 1, thus significantly associated with diagnosis of osteoporosis (*p* < 0.001 and *p* < 0.01). However, the association between osteoporosis and cardiovascular diseases, type 2 diabetes, and hypertension was revealed as not statistically significant after adjustment for age, physical activity, and the use of drugs known to be associated with bone demineralization. On the contrary, obesity and type 1 diabetes were confirmed to be associated with osteoporosis, even after adjustment for age, physical activity, and the use of osteoporosis-inducing drugs, with OR (confidence interval (CI) 95%) of 1.39 (1.05–1.83) and 1.46 (1.20–1.78), respectively.

All patients were divided into five groups corresponding to different BMI classes: subjects with optimal weight (BMI < 25), overweight people (BMI ≥ 25 but <30), grade 1 obesity (BMI ≥ 30 but <35), grade 2 obesity (BMI ≥ 35 but <40), and grade 3 obesity (BMI ≥ 40). The average age, BMI, AD-SoS measurements, and clinical characteristics for all five groups are shown in Table 3. A significant positive linear trend (*p* < 0.0001) was found across categories of BMI in the frequency of type 1 diabetes, cardiovascular diseases, hypertension, and dyslipidemia.

We investigated the ORs of osteoporosis for all five BMI groups, calculating both the crude and the ORs adjusted for age, physical activity, and the use of osteoporosis-inducing drugs, using the class of optimal weight as a reference group, with assigned value = 1 (Table 4). Considering the adjusted ORs, we found a significant linear trend over BMI categories, with a significant score test for trend (*p* < 0.001).

## 4. Discussion

The study took place in Salento, a sub-region of Southern Apulia characterized by the same ageing index of the entire Italian population and therefore representative of the general population, and will be particularly useful for further analyses when the registry becomes larger.

Though the majority of people enrolled in the registry was living in the area between Brindisi and Taranto (officially classified as “site of national interest” for environmental pollution related to the big steel and power plants of these two industrial cities), the effects of comorbities, attributable to environmental causes, on the proportion of secondary osteoporosis were not clear from our analysis of our database. 

The value of this cross-sectional study, carried out on 4909 postmenopausal women, consists in having demonstrated an independent association between obesity or diabetes and osteoporosis as diagnosed by using a radiation-free, non-invasive methodology such as QUS. This kind of technology has shown no significant differences when compared to DXA in terms of ability to predict hip fractures in large cohorts of patients or discriminate fractured patients. In particular, some authors found that QUS parameters (AD-SoS and UBPI) were able to identify subjects with a BMD *T*-score below 2.5 and those with a BMD *T*-score above the threshold value so that a correlation between ultrasound and densitometric results was moderate but positive [21].

DXA measurements were available for 303 subjects involved in this study, so we calculated the Pearson correlation between AD-SoS and BMD and found a positive correlation (*r* = 0.32; *p* < 0.001). We demonstrated in another study the ability of QUS to detect thalassemic patients with prevalent fragility fractures, and the results are comparable to those of DXA [22].

Correlation analysis between BMI and AD-SoS revealed a negative correlation coefficient, demonstrating a negative impact of BMI increase on bone ultrasound velocity at proximal phalanges. These findings seem to confirm the results of a cross-sectional study which found an association between fat mass and osteoporotic fractures [23]. The Pearson correlation coefficients of the BMD and BMI in the small sample of 303 subjects was 0.15 (*p* < 0.001). Although correlation was weak, it reflects the negative trend found between BMI and AD-SoS. 

The relationship between osteoporosis and obesity is currently controversial. Several evidences, including those from the NORA study, reported that an increasing BMI is associated with a BMD increase [24]. These observations could be explained taking into account the mechanical load exerted by the increased body weight, which results in an increase of bone mineral density [25]. Moreover, adipocytes in postmenopausal women represent the main source of estrogens and are known for their inhibitory activity of osteoclast-mediated bone resorption [26]. Finally, obesity is often related to high plasma insulin levels, a fact which contributes to the overproduction of sex hormones—estrogens and androgens—responsible for increased osteblast activity and reduced osteoclast activity [27].

On the contrary, other evidences suggest that obesity might negatively influence bone health [28,29]. This complex relationship between obesity and BMD could be explained by the effect on bone of a series of adipokines and cytokines secreted by adipose tissue, such as leptin, resistin, adiponectin, interleukin 6, and tumor necrosis factor-alpha [30,31]. One study involving obese patients found a lower lumbar spine BMD than was expected for age and BMI [32]. After performing pQCT (peripheral quantitative computed tomography) to analyze volumetric BMD, bone mineral content, and geometric and bone strength properties, some authors concluded that adiposity has a negative effect on trabecular and cortical bone [33].

These findings are in agreement with our results about the correlation between phalangeal QUS parameter and BMI.

One limitation to the explanation of our results could be the interference of soft tissue during the execution of phalangeal QUS. In several studies performed using QUS, soft tissues have been proven to reduce the speed of sound (SOS) transmitted across bones [33]; on the basis of these studies, some authors have proposed that the negative impact of BMI on QUS parameters can be ascribed to the interference of soft tissues [34]. In their paper, Biino et al. also found a negative correlation between AD-SoS and BMI, with AD-SoS showing the highest correlation coefficient with BMI among all QUS parameters; these authors, by bioimpedance analysis, proved that an increase in fat mass results in a negative impact on bone health [34]. On the basis of these findings and of the pQCT results, we accepted the appropriateness of QUS measurements in obese subjects.

According to the results of our study, the ORs of being osteoporotic for obese and diabetic subjects indicate that being obese or suffering from type 1 diabetes increases the probability of belonging to the osteoporotic group by 46% and 15%, respectively. For type 2 diabetes subjects, OR is not statistically significant after adjustment.

When stratifying our study population by class of BMI, the ORs of being osteoporotic have confirmed that an increase in the BMI value negatively affects bone mineralization. As shown in Table 4, when considering optimal weight (BMI < 25 kg/m^2^) as a reference group, we find a two-fold probability of being osteoporotic in subjects with grade 2 and grade 3 obesity (ORs = 1.94, CI: 1.27–2.97, and OR = 2.51, CI: 1.38–4.56, respectively). 

Our findings are consistent with results from other studies also concerning the association between diabetes and bone demineralization, where type 1 diabetes has already been associated with BMD reduction [35]; in people with type 2 diabetes, a higher BMD at the hip has been documented only at the baseline, with a rapid bone loss having been observed over time in a longitudinal study [36]. Leslie et al. observed that type 2 diabetes is associated with high BMD values and an increased risk of fragility fractures, thus claiming new markers and new preventive approaches to evaluate the risk of fractures in these patients [36]; these authors have also suggested including type 2 diabetes in the questionnaire of the FRAX international algorithm for the fracture risk assessment. A recent study carried out on Canadian women affected by type 2 diabetes confirmed the existence of a “bone fragility paradox”: although patients with type 2 diabetes had a normal femoral neck BMD compared to controls, they showed a weaker response to mechanical loading on the neck of the femur when entering a simulated mechanical model depicting forces acting on the femoral bone [37]. As hyperglycemia itself is an important factor in the regulation of osteoclast-mediated bone degradation, several studies have investigated the effect of hyperglycemia in fostering bone quality reduction and microarchitecture impairment in diabetic patients [37]. Moreover, non-enzymatic glycation (NEG), which consists of spontaneous reactions between extra-cellular sugars and free amino groups of several matrix proteins including collagen type I, leads to formation of molecular crosslinks which are known as advanced glycation end-products (AGEs). High concentrations of AGEs are known to increase bone fragility [38].

Phalangeal QUS was found to be comparable to DXA in discriminating people at higher risk of fracture, with QUS able to provide additional information for the skeletal assessment in type 2 diabetic patients [39]. As previous shown for obese patients, also about type 2 diabetes, there are evidences based on high resolution peripheral quantitative tomography (HR-pQCT) that demonstrated increased cortical porosity in the tibia and other bone sites of postmenopausal women suffering from type 2 diabetes [40].

Considering this ability of QUS to provide the physician with additional information on bone micro-architecture, this technology could be tested for specific use in clinical practice in combination with spinal deformity index (SDI), an index of bone quality used for the diagnosis of osteoporosis in subjects affected by type 2 diabetes. In a recent controlled study involving subjects with type 2 diabetes, spinal deformity index revealed its ability to discriminate subjects with vertebral fractures in a more specific way than the simple use of BMD for the diagnosis of osteoporosis [41]. 

Other limitations affect our study, such as the lack of data about the cortical and trabecular mineralization that could give us the entity of correlation between our data and bone status. This was not possible; since our study was conducted on such a large sample, a massive measurement with pQCT was not practical. Moreover, data about fat and lean mass are not available, and BMD measurements were only registered in a restricted section of the sample.

## 5. Conclusions

In conclusion, our study confirmed the high impact of obesity and type 1 and type 2 diabetes on osteoporosis.

## Figures and Tables

**Table 1:** Baseline characteristics of postmenopausal women stratified by bone mineralization status.

Variables	Osteoporotic	Osteopenic	Normal	*p*-Value
*N*	1306	3064	539	
Age, years	70.3 ± 8.6	62.8 ± 8.3	54.9 ± 7.1	<0.001
Weight, kg	71.2 ± 12.9	69.9 ± 12.4	66.9 ± 12.3	<0.001
Height, cm	1.55 ± 0.07	1.57 ± 0.07	1.59 ± 0.06	<0.001
BMI (kg/cm^2^)	29.84 ± 5.56	28.64 ± 5.19	26.71 ± 4.87	<0.001
AD-SoS, (m/s)	1842.2 ± 60.7	1967.5 ± 46.5	2087.3 ± 44.1	<0.001
Phalangeal *T*-Score	−4.22 ± 0.92	−2.17 ± 0.59	−0.43 ± 0.50	<0.001
Smoking, yes (%)	79 (6.09)	282 (9.23)	24 (13.81)	<0.001
Alcool, yes (%)	389 (29.78)	774 (25.26)	108 (20.12)	<0.01
Hip fractures, *n* (%)	45 (3.46)	34 (1.12)	2 (0.30)	<0.001
Vertebral fractures, *n* (%)	29 (4.02)	17 (0.55)	3 (0.90)	<0.01
Wrist fractures, *n* (%)	48 (6.65)	43 (2.53)	2 ( 0.60)	<0.001
Other fractures, *n* (%)	109 (15.10)	142 (8.35)	5 (1.5)	<0.001
Parental fractures, *n* (%)	159 (12.19)	499 (16.28)	97 (18.02)	<0.005
Obesity, *n* (%)	565 (43.35)	1082 (35.33)	110 (20.42)	<0.001
Hypertension, *n* (%)	722 (55.26)	1449 (47.38)	178 (33.03)	<0.001
Type 1 Diabetes	54 (14.96)	46 (1.5)	6 (1.11)	<0.001
Type 2 Diabetes	138 (10.57)	239 (7.8)	21 (3.9)	<0.001
Dislipidemia, *n* (%)	264 (20.22)	621 (20.28)	50 (9.31)	0.001
Cardiovascular diseases, *n* (%)	146 (11.22)	236 (7.70)	24 (4.50)	<0.001
Rheumatic diseases, *n* (%)	84 (6.37)	144 (4.70)	27 (5.11)	0.238
Previous or current use of inducing-osteoporosis drugs, *n* (%)	233 (17.87)	319 (18.75)	118 (21.92)	0.285
Previous or current use of antifracturative drugs, *n* (%)	541 (41.41)	827 (27.04)	87 (16.22)	<0.001
Moderate physical activity, yes (%)	398 (30.47)	1275 (41.68)	230 (42.64)	<0.001
Regular physical activity, yes (%)	13 (0.97)	104 (3.43)	29 (5.41)	<0.001
Total physical activity, yes (%)	398 (30.5)	1275 (41.6)	232 (43.11)	<0.001

AD-SoS: amplitude-dependent speed of sound.

**Table 2:** Odds ratios (ORs) and 95% CIs of osteoporosis (AD-SoS *T*-score lower than −3.2 SD) in presence of diabetes, cardiovascular diseases, hypertension, and obesity.

Variables	OR (95% CI)	*p*-Value	Adjusted OR ^1^ (95% CI)	*p*-Value
Type 1 Diabetes	2.94 (2.00–4.33)	<0.001	2.49 (1.63–3.82)	<0.01
Type 2 Diabetes	1.52 (1.22–1.89)	<0.001	1.15 (0.90–1.46)	0.229
Cardiovascular diseases	1.63 (1.23–2.17)	<0.01	0.93 (0.67–1.28)	0.645
Hypertension	1.51 (1.27–1.79)	<0.001	0.95 (0.78–1.15)	0.596
Obesity	1.56 (1.31–1.86)	<0.001	1.46 (1.20–1.78)	<0.001

^1^ ORs were adjusted for age, physical activity, and the use of osteoporosis-inducing drugs.

**Table 3:** Baseline characteristics of study population stratified by classes of BMI.

Variable	Healthy Weight	Overweight	Grade 1 Obesity	Grade 2 Obesity	Grade 3 Obesity
	(BMI < 25)	25 ≤ BMI < 30	30 ≤ BMI < 35	35 ≤ BMI < 40	BMI ≥ 40
*N*	1367	1931	1088	396	127
Age (years) ^1^	61.6 ± 9.8	64.3 ± 9.8	65.0 ± 8.8	64.7 ± 9.0	64.9 ± 8.2
Weight (kg) ^1^	57.05 ± 6.21	67.75 ± 6.50	77.56 ± 7.29	86.71 ± 8.03	102.50 ± 12.50
Height (m) ^1^	1.58 ± 0.06	1.57 ± 0.06	1.55 ± 0.07	1.53 ± 0.06	1.53 ± 0.06
BMI ( kg/m^2^) ^1^	22.75 ± 1.75	27.46 ± 1.44	32.03 ± 1.39	36.89 ± 1.42	43.62 ± 3.54
AD-SoS (m/s) ^1^	1980.9 ± 93.2	1947.3 ± 92.8	1939.3 ± 88.0	1916.6 ± 97.0	1921.3 ± 86.1
Hypertension (*n* (%)) ^1^	401 (29.4)	907 (47.0)	627 (57.7)	260 (65.9)	97 (76.5)
Diabetes type 1 and 2 (*n* (%)) ^1^	76 (5.6)	164 (8.5)	147 (13.6)	85 (21.5)	31 (24.5)
Type 1 Diabetes (*n* (%)) ^1^	11 (0.80)	29 (1.50)	39 (3.58)	19 (4.80)	8 (6.30)
Type 2 Diabetes (*n* (%)) ^1^	51 (3.73)	149 (7.72)	121 (11.12)	5 (14.90)	18 (14.17)
Dislipidemia, (*n* (%)) ^1^	192 (14.1)	378 (19.6)	230 (21.2)	87 (22.0)	31 (24.5)
Cardiovascular diseases (*n* (%)) ^1^	76 (5.6)	144 (7.5)	109 (10.1)	44 (11.2)	19 (15.3)
Rheumatic diseases (*n* (%)) ^2^	84 (6.2)	93 (4.83)	52 (4.8)	18 (4.7)	7( 6.1)

^1^ *p* < 0.0001. ^2^ *p* value not statistically significant.

**Table 4:** Crude and adjusted odds ratios and 95% CIs of osteoporosis for different BMI categories (optimal weight was chosen as a reference group, with assigned value = 1).

BMI Category	OR ^1^	*p*-Value	Adjusted OR ^1,2^	*p*-Value
Optimal weight	1		1	
Overweight	1.52 (1.2–1.93)	<0.001	1.22 (0.93–1.6)	0.151
Grade 1 obesity	1.85 (1.43–2.39)	<0.0001	1.4 (1.05–1.87)	<0.05
Grade 2 obesity	2.34 (1.67–3.27)	<0.0001	1.94 (1.27–2.97)	<0.01
Grade 3 obesity	2.76 (1.75–4.35)	<0.0001	2.51 (1.38–4.56)	<0.01

^1^ Significant linear trend with increasing BMI category, resultant *p* value of the score test for trend was <0.0001. ^2^ ORs adjusted for age, physical activity, and the use of drugs causing osteoporosis.
